# Detecting Proteins in Highly Autofluorescent Cells Using Quantum Dot Antibody Conjugates

**DOI:** 10.3390/s90907540

**Published:** 2009-09-23

**Authors:** Karen M. Orcutt, Shanshan Ren, Kjell Gundersen

**Affiliations:** Department of Marine Science, University of Southern Mississippi, 1020 Balch Blvd., Stennis Space Center, MS 39529, USA; E-Mails: Shanshan.Ren@usm.edu (S.R.); Kjell.Gundersen@usm.edu (K.G.)

**Keywords:** quantum dot, autofluorescent cells, nitrogen fixation, iron stress, cyanobacteria

## Abstract

We have applied quantum dot (Qdot) antibody conjugates as a biomolecular probe for cellular proteins important in biogeochemical cycling in the sea. Conventional immunological methods have been hampered by the strong autofluorescence found in cyanobacteria cells. Qdot conjugates provide an ideal alternative for studies that require long-term imaging of cells such as detection of low abundance cellular antigens by fluorescence microscopy. The advantage of Qdot labeled probes over conventional immunological methods is the photostability of the probe. Phycoerythrin bleaches in cyanobacterial cells under prolonged UV or blue light excitation, which means that the semiconducting nanocrystal probe, the Qdot, can yield a strong fluorescent signal without interference from cellular pigments.

## 1. Introduction

Detecting proteins by epifluorescence microscopy in whole-cells of cyanobacteria has been problematic and sometimes impossible due to the presence of highly autofluorescent natural pigments [1–3]. This strong autofluorescence from natural pigments, such as phycoerythrin, can mask probes consisting of organic fluorophores. Due to the low photostability of organic fluorophores, bleaching of natural pigments to yield fluorescent probe signals has been very difficult. In order to avoid phycorerythrin autofluorescence, fluorochromes in the blue region have been used for filamentous cyanobacteria [4] but unicellular cyanobacteria have also been found to exhibit a blue autofluorescence rendering this spectral region useless for reporter probes as well [1,5, pers. ob.]. Here we circumvent these limitations by reducing natural autofluorescence by exposure to blue or UV light prior to viewing and by applying a recent advance in nanotechnology. Semiconducting nanocrystals or quantum dots (Qdots) can be conjugated to antibody probes and in this study we use this technology to detect proteins *in situ* in highly autofluorescent unicellular and filamentous cyanobacteria. Qdots (CdSe/ZnS) exhibit a size-dependent tunable bright photoluminescence that spans the visible spectrum. As a consequence, Qdots provide an ideal alternative for studies that require long-term and multicolor imaging of cells such as detection of low abundance cellular antigens by fluorescence microscopy.

Assessing the physiology of autofluorescent cells using the whole-cell approach has been very difficult due to limitations with conventional fluorophores. In this study, we describe a physiological stress experiment to highlight the use of Qdot probes for targeting a protein involved in an iron stress response in autofluorescent cyanobacterial cells. The cell protein, IdiA (iron deficiency induced protein A), is a 34 kDa protein in cyanobacteria that is required for growth under iron stress conditions [6]. IdiA is localized intracellularly usually associated with the thykaloid or periplasmic membrane in *Synechococcus* and *Synechocystis* strains [7,8]. Recently, IdiA has been found in open ocean cyanobacteria *Synechoccocus*, *Trichodesmium* and *Crocosphaera* (*Synechocystis*) and is considered an indicator of iron stress [9]. Detection of iron stress using IdiA in microplankton requires bulk protein extraction and analysis by Western blotting [9]. In this paper, we describe the use of Qdot conjugate probes in whole-cell detection of IdiA in iron stressed unicellular and filamentous cyanobacterial cells.

A recent improvement for immunodetection in cyanobacterial cells used whole-cell immunocytochemical detection to improve protocols for highly fluorescent unicellular and filamentous cyanobacterial cells [1–3]. This method uses a primary antibody probe combined with a secondary antibody conjugated to horseradish peroxidase followed with a signal amplification step. These studies used the protein nitrogenase, as the cellular target in the immunocytochemical assay. Nitrogenase is an essential enzyme in biological nitrogen fixation, which has been found to be a significant biogeochemical process in the marine environment. Immunofluorescence detection was a first choice in the Taniuchi *et al*. study [1], but the highly autofluorescent cells limited the use of this technique.

Microbial unicellular nitrogen fixers have been reported to be a significant player in the nitrogen cycle of the upper ocean [10,11]. In the North Pacific Ocean, unicellular diazotrophs can support a significant amount of new production in oligotrophic waters equal to 10% of global oceanic new production [11]. Therefore, it would be advantageous to detect single cell nitrogen fixers in the environment. In this paper, we describe the use of Qdot antibody conjugates for detection of nitrogenase in highly autofluorescent unicellular cyanobacteria *Crocosphaera watsonii* (WH8501) and in the filamentous cyanobacteria *Trichodesmium erythraeum* (IMS101) cells. This nanotechnology allows whole-cell detection of physiologically relevant proteins in highly autofluorescent cyanobacterial cells.

## 2. Experimental Section

### 2.1. Culture Conditions

Laboratory cultures of the *C. watsonii* strain (WH8501) were grown in SO and SN media [12; with and without combined nitrogen] in a 12:12 light dark cycle at 28 °C. Cultures of the *T. erythraeum* strain (IMS101) were grown on YBC11 media [13] in a 12:12 light dark cycle at 28 °C. The cultures were grown in either iron deplete or iron replete conditions. The iron deplete cultures had iron removed from the trace metal mix in order to induce accumulation of the iron stress protein IdiA.

### 2.2. Immunolabeling Procedure

Cells of *C. watsonii* (2 mL) were immunolabeled using a modified method derived from [14,15]. The single cell cyanobacteria were collected by filtration on a 1.2 μm filter and placed into ice cold 95% ethanol at −20 °C overnight. The *C. watsonii* cells were always collected in the middle of the dark period to ensure maximum expression of the nitrogenase protein. Cells were centrifuged and fixed in 1% paraformaldehyde (pH 6.8, buffered with 100 mM PBS) at room temperature for 15 min, and incubated in lysozyme (10 mg/mL in 100 mM Tris-HCL, 20 mM EDTA, pH 8.0, Sigma) for 1 h at 37 °C, followed by achromopeptidase (60 U/mL in 50 mM Tris-HCL, pH 7.0, Sigma) for 30 min at 37 °C to permealize the cells. The permealization method was tested on centrifuged cell suspensions with the Bac/Light Live Dead stain (Invitrogen). Positive results (red stained cells) were obtained showing the integrity of the membrane had been perforated. The cells were blocked in 3% bovine serum albumin (BSA, Sigma) for 1 h at room temperature. After blocking, the cells were incubated in polyclonal primary antibody solution rabbit IgG (anti-IdiA or anti-nitrogenase) at a titer of 1:100 for 1 h at room temperature. The cells were then incubated with secondary Qdot 525 or Qdot 605 goat anti-rabbit IgG (Invitrogen) 1:100 solution overnight at 4 °C. After each antibody incubation, the cells were rinsed once with 0.05% Tween in PBS followed by a rinse in PBS. Cells were incubated in Qdot 525 or Qdot 605 goat anti-rabbit IgG 1:100 solution without primary antisera as a control for non-specific binding. Each step unless indicated in the procedure, had three rinses with PBS. Cells grown in SN media were used as a control. Filaments of *T. erythraeum* were collected by filtration (3 mL) on a 2 μm filter and suspended in ice cold 95% ethanol at −20 °C overnight. The filaments were collected on a 2 μm filter, placed in a plastic Petri dish and the filter encircled with a PAP pen (Energy Beam Sciences, Inc.). The filaments were permeabilized with lysozyme (10 mg/mL) for 3 min at 37 °C followed by 0.5% dimethyl sulphoxide (DMSO, Sigma) for 15 min at 4 °C. The *Trichodesmium* filaments were blocked in 3% BSA for 1 h prior to incubation in 1:100 titer primary anti-nitrogenase or anti-IdiA sera for 4 h at room temperature. Finally, the filaments were incubated in secondary 525 Qdot goat anti-rabbit IgG sera overnight at 4 °C. The *C. watsonii* cells were photographed immediately to document autofluorescence and then again after exposure to blue light (450–480 nm) for 60 s using an Olympus BX51 fluorescent microscope equipped with a DP71 12 bit color digital camera and software. The *T. erythraeum* filaments however, required an additional pretreatment of 4–6 hour exposure to short wave UV to reduce autofluorescence before blue light exposure for 15 min. All preparations were viewed using an Olympus U-MWB2 epifluorescent microscope with a wide blue filter cube with excitation at 450–480 nm, dichroic mirror 500 nm and longpass barrier filter at 515 nm.

## 3. Results and Discussion

### 3.1. Detecting in situ Iron Stress with Qdot Antibody Conjugates

**Figure 1.**
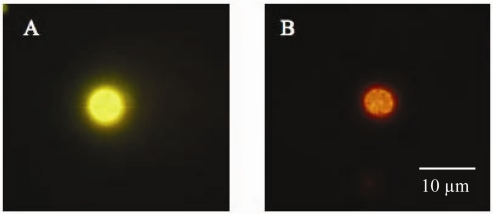
Immunofluorescence detection of IdiA accumulation in an autofluorescent *C. watsonii* cell labeled with Qdot 605 secondary antibody conjugate against the IdiA primary antibody prior to photobleaching (A). The same field after photobleaching exhibiting strong Qdot 605 labeling. Photograph enlarged for detail.

**Figure 2.**
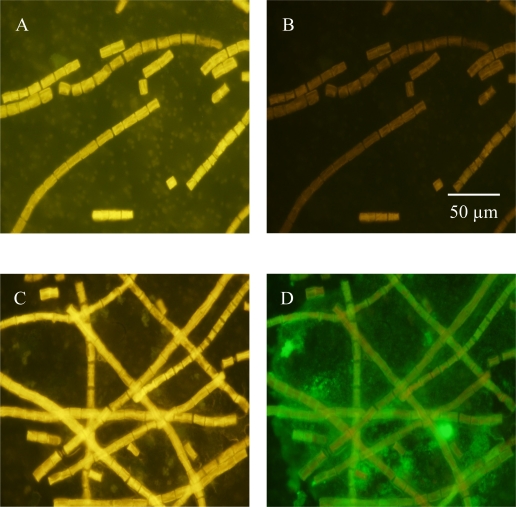
Immunofluorescence detection of IdiA accumulation in *T. erythraeum* cells. Binding of the IdiA antibody to cellular structures was visualized by subsequent incubation with the Qdot 525 anti-rabbit IgG antibody. Autofluorescent trichomes from iron replete cultures prior to blue light photobleaching treatment (A). The same field after photobleaching with minimal Qdot 525 signal (B). Autofluorescent trichomes from iron depleted cultures prior to photobleaching treatment (C). The same field after photobleaching exhibiting strong Qdot 525 labeling of the trichomes and cellular debris (D). All images captured at 1000X magnification.

Iron stressed *C. watsonii* cells probed with the IdiA antibody and labeled with Qdot 605 secondary antibody probe that were exposed to blue light for 60 s displayed a bright fluorescent signal from the photostable nanocrystal conjugate (Figure 1B). *T. erythraeum* trichomes grown in iron replete media did not show any labeling of IdiA protein (Figure 2A,B) following 4 h exposure to short wave UV and subsequent 15 min exposure to blue light. *T. erythraeum* trichomes grown on iron deplete media showed IdiA labeling along the trichomes in many cells (Figure 2C,D) following the same pretreatment as in iron replete cultures. Iron stressed trichomes were associated with cellular debris or exudates that incorporated some of the Qdot antibody conjugate in the extracellular matrix (Figure 2C,D). Omitting the primary antibody showed no labeling in *T. erythraeum* cells (data not shown). This fluorescent immunolabeling technique would be impossible with conventional organic fluorophores as the natural autofluorescence (Figure 1A, Figure 2A,C) would mask the antibody signal. Also, organic fluorophores rapidly fade under blue light exposure and especially prolonged exposure to short wave UV. Thus, the photostability of Qdot antibody probes allows for unprecedented fluorescent immunodetection in highly autofluorescent cells. The interference of the natural blue fluorescence found in unicellular cyanobacteria [1,5] was avoided by using the Olympus WB2 wide blue filter cube with emission greater than 515 nm. Unlike conventional organic fluorophores, Qdots have a wide absorption spectrum, a narrow, tunable emission spectrum, and are photo-chemically stable. The unique optical properties of Qdots make them ideal probes for highly autofluorescent cyanobacterial cells.

**Figure 3.**
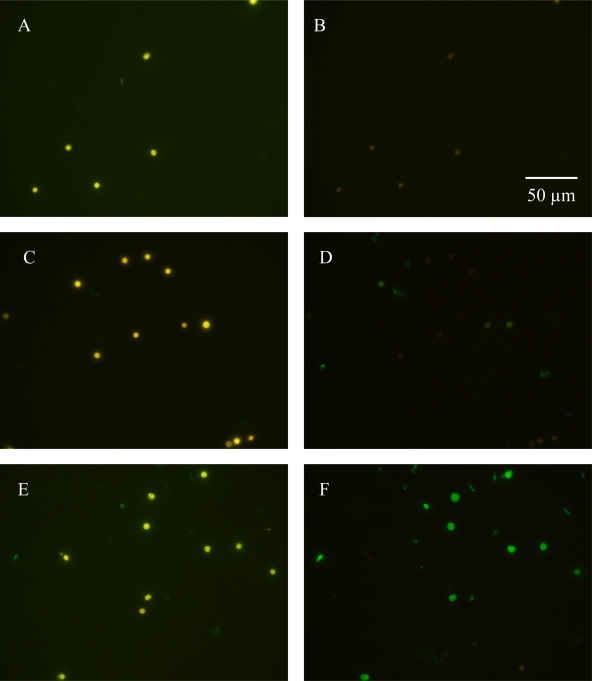
Immunodection of IdiA accumulation in *C. watsonii* cells. Binding of the IdiA antibody to cellular structures was visualized by subsequent incubation with the Qdot 525 anti-rabbit IgG antibody. Autofluorescent cells from iron deplete cultures where the primary anti-IdiA antibody was omitted prior to blue light photobleaching treatment (A). The same field after photobleaching with no Qdot 525 signal (B). Autofluorescent cells from iron replete cultures prior to photobleaching treatment (C). The same field after photobleaching with minimal Qdot 525 labeling (D). Autofluorescent cells from iron depleted cultures prior to photobleaching treatment (E). The same field after photobleaching exhibiting strong Qdot 525 labeling of the cells (F). All images captured at 1000X magnification.

Cells of *C. watsonii* did not show any non-specific binding of the Qdot conjugate when the primary antiserum was omitted (Figure 3A,B). Cells grown in iron replete media only showed trace labeling of IdiA (Figure 3C,D) while cells grown in iron deplete media showed the most pronounced labeling (Figure 3E,F). This whole-cell approach can detect iron stress, expressed as IdiA, in single cells and filaments of cyanobacteria providing a useful and powerful probe for physiology studies of open ocean cyanobacteria.

### 3.2. Detecting Nitrogenase in Highly Autofluorescent Cells

**Figure 4.**
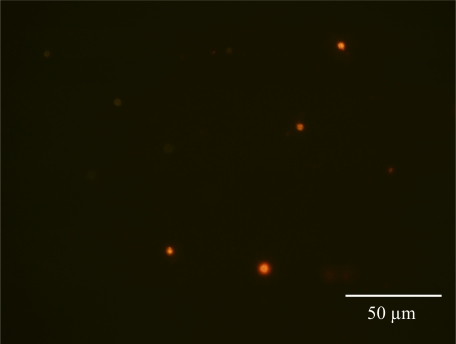
Immunofluorescent detection of nitrogenase in *C. watsonii* WH8501 cells. Binding of the nitrogenase antibody to cellular structures was visualized by subsequent incubation with the Qdot 605 anti-rabbit IgG antibody. Photograph captured at 1000X magnification after 60 s exposure to blue light.

**Figure 5.**
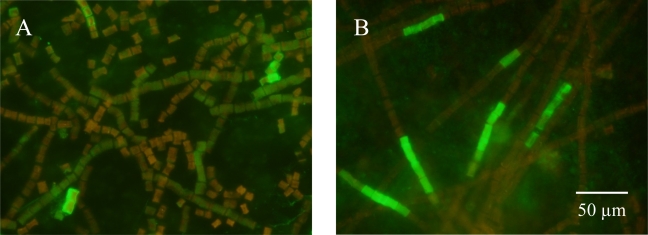
Immunofluorescent detection of nitrogenase in *T. erythraeum* cells. Binding of the nitrogenase antibody to cellular structures was visualized by subsequent incubation with the Qdot 525 anti-rabbit IgG antibody. *T. erythraeum* cells from a 35 day old culture (A) and from a 55 day old culture (B). Both images are captured at 1000X magnification.

Up until now, fluorescent immunolabeling of nitrogenase in cyanobacteria has been problematic due to autofluorescence [1–3,5]. Additionally, the use of fluorescent probes in unicellular cyanobacteria are frequently masked by a natural blue fluorescent pigmentation [1,5]. Due to its photostability, broad absorption spectrum and tunable emission pattern, Qdot antibody probes can overcome these issues. Nitrogenase was immunolabeled with Qdot conjugate 605 in *C*. watsonii (Figure 4) and Qdot conjugate 525 in *T. erythraeum* (Figure 5A,B). The *C. watsonii* cells were exposed to blue light for 60 s to reduce autofluorescence. *Trichodesmium* filaments however, took longer to photobleach the natural pigments and required additional hours of short wave UV exposure to reduce autofluorescence. In most cases, extended exposure to UV light would photobleach conventional organic fluorophores, but the Qdot antibody conjugate yielded a bright luminescent signal. Cells of *C. watsonii* grown in SN medium did not show significant labeling (data not shown). All cyanobacterial cells incubated in Qdot secondary antibody solution without primary antibody to nitrogenase did not show any labeling with Qdot conjugates (data not shown). This is the first report of whole-cell immunolocalization of nitrogenase and IdiA using Qdot conjugates in both filamentous and single cell cyanobacteria.

Two different labeling patterns were observed in *T. erythraeum* cells. Figure 5A shows a pattern that is similar to recent work using an improved immunocytochemical technique [2,3] and immungold labeling [20]. This pattern showed labeling of nitrogenase that was less intense (relative to Figure 5B), more diffuse and appeared in a greater number of cells along the trichome (Figure 5A). The pattern in Figure 5B showed intense labeling of nitrogenase in a subset of cells along the trichome. This pattern is similar to previous work using Alexa-350 fluorochromes [4,16,17] and to studies using immunogold labeling [18,19]. The difference in labeling pattern might be attributed to age or physiology as suggested by Ohki and Taniuchi [3] since the cells in Figure 5B were 20 days older than the cells in Figure 5A. The trichomes in Figure 5B had formed bundles in culture while the trichomes in Figure 5A were free trichomes. Bundle formation may be a precursor to consolidation of the enzyme in a subset of cells. The use of Qdot antibody conjugates in this study clearly demonstrates the usefulness of these fluorophores in detecting low abundant cellular antigens as well as intense labeling of filaments in highly autofluorescent cyanobacteria such as *T. erythraeum*.

## 4. Conclusions

Qdot antibody conjugates provide an ideal probe for detecting intracellular proteins in highly autofluorescent cyanobacterial cells after exposure to UV or blue light. Antibodies conjugated to Qdots have been used to detect proteins in marine eukaryotic phytoplankton [21]. Here we provide the first application of this novel technology in highly autofluorescent cells in order to detect iron stress (IdiA) and the presence of nitrogenase in whole-cells of filaments and single cells of prokaryotic cyanobacteria. The photostability of Qdot antibody probes allows for unprecedented fluorescent immunodetection in highly autofluorescent cells.
